# Proximal femur fat fraction variation in healthy subjects using chemical shift-encoding based MRI

**DOI:** 10.1038/s41598-019-56611-8

**Published:** 2019-12-27

**Authors:** Pedro Augusto Gondim Teixeira, Tanguy Cherubin, Sammy Badr, Adrien Bedri, Romain Gillet, Eliane Albuisson, Alain Blum

**Affiliations:** 1 Guilloz imaging department, Central Hospital, University Hospital Center of Nancy, 29 avenue du Maréchal de Lattre de Tassigny, 54035 Nancy, cedex France; 2 Department of Radiology and Musculoskeletal Imaging, CHRU Lille Centre de Consultations et d’Imagerie de l’Appareil Locomoteur, 59037 Lille, France; 3 CHRU-Nancy, DRCI, Département MPI, Unité de Méthodologie, Data management et Statistique UMDS, F-54000 Nancy, France

**Keywords:** Diagnostic markers, Prognostic markers

## Abstract

The objective of this study *was* to describe the normal variation of bone marrow fat content in the proximal femur considering the influence of side, age, sex and body mass index using fat fraction MRI. From September 2012 to July 2016, the MRI of 131 patients (258 hips) considered to have a normal MRI appearance were retrospectively evaluated. Patient records were searched to allow calculation of the body mass index (BMI). Water-fat based chemical shift MRI was available for all patients included. Proton density fat fraction maps were calculated, and measurements were performed in the femoral epiphysis, intertrochanteric region, and greater trochanter. The influence of patient age, sex, hip side and BMI on fat fraction values was assessed. Fat fraction was significantly different in the different locations evaluated (*P* = 0.0001). Patient sex and age significantly influenced fat fraction values in all regions evaluated (*P* < 0.02) with the exception of the epiphysis for sex (p = 0.07). In all locations, PDFF values were higher in men compared to women (3.3%, 4.4% and 13.1% higher in the epiphysis, greater trochanter and intertrochanteric region respectively). The intertrochanteric region presented the lowest fat fraction values with the highest variation compared to the greater trochanter and the epiphysis. BMI only influenced fat fraction values in the intertrochanteric region of females over 42 years old (P = 0.014). The interobserver variability of the measurements performed was considered to be excellent (ICC = 0.968). In conclusion, patient sex, age, and measurement location significantly influenced fat fraction values indicating that specific standards of reference are needed depending on these factors.

## Introduction

Various studies have highlighted the relationship between bone marrow fat content and bone mineralization, suggesting a potential use of bone marrow fat content estimation with magnetic resonance imaging (MRI) for osteoporosis assessment^1,2^. An accurate quantitative appreciation of bone marrow adiposity could also be a promising imaging biomarker for the evaluation of many conditions, such as Gaucher’s disease, myelofibrosis or tumour infiltration^3–7^. Alcohol and corticosteroid intake could also affect bone marrow adiposity and are considered as risk factors for avascular osteonecrosis of the femoral head^8^. Bone marrow fat quantification on MRI can be achieved by two means: MR single-voxel proton spectroscopy and chemical shift encoding-based water-fat imaging (WFI)^9^. Proton spectroscopy is the gold standard for the quantification of marrow fat content, but the technique is complex, time-consuming and measurements can be influenced by various factors such as magnetic field inhomogeneities, the presence of calcium and tissue architecture^10^. Moreover, it is not possible to generate fat fraction maps based on proton spectroscopy^9^. WFI is mainly derived from Dixon techniques and has recently benefited from many technical advances in liver imaging for quantitative fat assessment^11–13^. Iterative decomposition of water and fat with echo asymmetry and least-squares estimation (IDEAL) sequences emerged from these developments, and have been demonstrated to be a robust method for fat content estimation with the advantages of being fast and widely available. By taking into account T_2_ decay effects and a multipeak spectral model of fat, this technique allows the generation of proton-density fat fraction (PDFF) maps and is an interesting option for bone marrow fat content assessment^14–16^.

Since the bone marrow can be considered an active endocrine organ, various factors, such as age, sex, and body habitus might affect the bone marrow fat content^17^. Thus, determining the normal variation of bone marrow fat content is an essential step for the clinical application of WFI. Recently, patient sex and age have been shown to influence vertebral bodies fat content in using WFI^18,19^. The proximal femur constitutes an evaluation target in various clinical scenarios such as fracture risk evaluation in osteoporosis, tumour burden assessment and follow-up of marrow deposition disorders^20–22^. Although the influence of these factors on marrow fat content has been assessed in de femur with proton spectroscopy, there is scarce information about the normal variation of femoral fat marrow content with WFI^23^. The lack of valid referential hampers the clinical application of WFI for bone adiposity quantification in the proximal femur.

In this study, we sought to describe the normal variation of bone marrow fat content using WFI in the proximal femur considering the influence of side, age, sex, and body mass index. This information could facilitate the clinical application of quantitative bone marrow adiposity assessment using WFI and serve as bases for future research.

## Materials and Methods

### Study population

From September 2012 to July 2016 MR images of 244 patients evaluated with WFI were retrospectively evaluated. Patients referred for the evaluation of unilateral or bilateral hip pain imaged on a 3 T MR scanner were included in this study. Due to the short sequence duration and the potential clinical impact, WFI is comprised in the standard hip imaging protocol in our institution since it became available in early 2012. As part of our institution’s imaging department registering procedure, all patients are asked to provide demographic information (sex, age, weight, and height). Height and weight were not directly measured. The body mass index (BMI) in kg/m^2^ was calculated (BMI = Weight/Height^2^). Among these patients, 146 presented no identifiable bone abnormalities on MRI. Twenty-nine hips (from 13 patients) were excluded due to magnetic susceptibility artefacts related to metallic bodies in the proximal femur (total hip arthroplasty or surgical osteosynthesis). Patients in which total arthroplasty or osteosynthesis was performed due to osteonecrosis or osteoporotic proximal femoral fractures were excluded even if the contralateral hip had a normal appearance on MRI. The study population was thus composed of 131 patients. BMI values were available for 108 of the patients included.

In our institution retrospective studies with fully anonymized patient data dot no require institutional review board (IRB) approval (Comission Nationale de l′Informatique et des Libertés - CNIL reference methodology MR003 2018-154). Thus, ethics approval was not sought and none of the researchers had access to identifying patient information when analysing study data.

### Image acquisition

All images were acquired with a 3 T MRI scanner (MR750w, GE Healthcare, Milwaukee, WI, USA) using a body coil. Both hips were imaged with conventional T2-weighted fat-saturated fast spin echo sequences in two different orthogonal planes and a coronal T1-weighted fast spin echo sequences. The following parameters were used for T2-weighted sequences: time repetition (TR) = 5482–9747 ms, time echoes (TE) = 38–110 ms, number of excitations (NEX) = 3–4, bandwidth = 22–40 kHz, echo train length (ETL) = 16. T1-weighted sequences were acquired with the following parameters: TR = 454–795 ms, TE = 9–14 ms, NEX = 1–3, bandwidth = 54 kHz, ETL = 3. For both conventional sequences, the slice thickness was 3.5 mm with a gap of 0.5 mm. The field of view (from 69 × 18 cm to 146 × 38 cm) and the acquisition matrix (from 320 × 320 to 416 × 320 pixels) were adapted to the patient’s body habitus.

Chemical shift-encoding based water-fat imaging was performed using a 3D spoiled gradient echo sequence with 12 echoes (iterative decomposition of fat and water with echo asymmetry and least-squares estimation – IDEAL). Images were acquired in the coronal plane with the following parameters: Flip angle 5° (to avoid T1 bias effects), number of shots 2, FOV 153 × 40 mm, matrix 256 × 160 mm, slice thickness 5 mm with no gap, bandwidth 125 kHz. R2* and B0 mapping are included in this sequence to allow B0 and T2* correction. A factor 2 parallel imaging acceleration with auto-calibrating reconstruction for cartesian sampling (ARC) was used. As a result of this acquisition, four image series were reconstructed automatically: in phase, out of phase, water only and fat only. Then, a pixel-by-pixel PDFF map was also calculated automatically in the MR scanner console using the following equation:

$$PDFF( \% )=fat\,only\,signal\,\ast \,100/(water\,only+fat\,only\,signal)$$

### Image analysis

A radiologist with 12 years of clinical experience with musculoskeletal images evaluated conventional images to identify the normally appearing proximal femurs in a PACS workstation (Fujifilm Synapse v4.1.600, Fujifilm, USA). Bone marrow with a high signal intensity and no signs of marrow replacement on T1-weighted images and homogeneous low signal intensity on T2-weighted fat-saturated sequences was considered to be normal. Small signal changes in the bone marrow related to areas with a high red marrow content (intermediate signal intensity on T2-weighted fat-saturated and discrete low signal intensity on T1-weighted images) were considered to be normal. Hips with lytic bone lesions or advanced femoro-acetabular degenerative joint disease were considered to be abnormal. Hip studies with soft-tissue changes such as gluteal, adductor or hamstrings tendinopathy without associated osseous signal anomalies were included in this study. After this analysis 131 patients were considered to have normally appearing hips. Six of these patients presented a unilateral total hip arthroplasty; however, the proximal femurs of the contralateral side were found to be normal. Thus 258 hips were available for evaluation. The other 156 patients were not included in the analysis.

**Figure 1:**
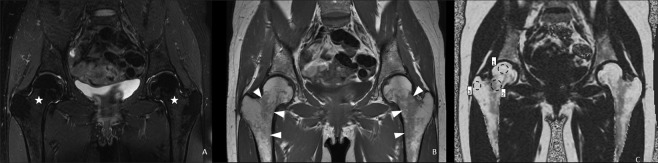
41-year-old female referred for the evaluation of chronic lower back and inguinal pain. (**A**,**B**) Coronal T2-weighted fat-saturated and T1-weighted images demonstrating a normal aspect of the proximal femora with a homogeneous low T2 signal (stars in A) and a high T1 signal with mottled areas of lower signal intensity corresponding to red marrow zones in the intertrochanteric region (arrowheads in **B**). (**C**) Proton density fat fraction map of the same patient demonstrating ROI positioning in the right femur. ROI 1, 2 and 3 are positioned in the femoral epiphysis (dashed circles), intertrochanteric region and greater trochanter respectively. Note that the ROI were placed strictly in the bone medullary cavity excluding cortical bone and peri-articular soft tissues.

Fat fraction estimation was performed on the same PACS workstation by a radiologist with three years of clinical experience with MRI by the following procedure (Fig. 1):

All images of the PDFF map were browsed to select the images depicting: the central portion of the femoral epiphysis; the central portion of the proximal femoral metaphysis (intertrochanteric region) and the central portion of the greater trochanter. Then one elliptical ROI measuring more than 100 mm² was placed in the center of each of these three zones. Care was taken to place these ROI entirely within the bone marrow excluding cortical bone or cartilage. The mean PDFF value (%) on the pixels contained in these ROI was considered in the analysis.

A third radiologist with two years of clinical experience with MRI used the same procedure to measure the PDFF of 50 randomly selected hips to allow interobserver reproducibility assessment. An ROI was also placed in the subcutaneous fat of all hips evaluated to serve as quality control.

### Statistics

Statistical analysis was performed with the IBM SPSS statistic software (V.22, Armonk, NY: IBM Corp). Qualitative data are presented as frequency (%). Because of a non-Gaussian data distribution, quantitative data are presented with median [Inter Quartile Range - IQR]. IQR/median(M) ratios were calculated to express the variation of PDFF values across the subgroups evaluated. The alpha risk was set to 0.05. The comparisons between PDFF values in the three locations (epiphysis, intertrochanteric region, and greater trochanter) evaluated were performed with the Friedman test. For each location, the influence of side, BMI, age and sex, on PDFF values were assessed with the Mann and Whitney test or correlations performed with the Spearman’s Rho. As the PDFF values have non-Gaussian distribution and are highly correlated, we did not use multivariate regression models. We chose a strategy allowing us to hierarchize variables and identify subgroups of subjects with respect to the sex, age, and BMI. A Chi-squared automatic interaction detector (CHAID) method was used for each PDFF values from the three locations studied. Two data sets (right and left hips) were analysed independently in order to compare the results and to evaluate their reproducibility and coherence. Only discriminations identified in both sides were represented. Intra-class correlation values (ICC) were calculated to assess the inter-observer agreement of the PDFF measurements performed. ICC values of 0–0.20 were considered to represent slight; 0.21–0.4, fair; 0.41–0.60, moderate; 0.61–0.80, good; 0.81–1, excellent agreement.

### Statistics and biometry

One of the authors (Professor Albuisson) has significant statistical expertise.

### Ethical approval

Institutional Review Board approval was not required because this was a retrospective studies based on the anonymised analysis of clinical images.

### Informed consent

Only if the study is on human subjects: Written informed consent was not required for this study because this was a retrospective study with full anonymised image analysis.

## Results

### Population

Among the 131 patients (258 hips) included the median age was 47 [36–60] years. There were 69 females with a median age of 49 [38–63] years and 62 males with a median age of 44 [34–57] years (M/F sex ratio of 1.1). The median BMI in the studied population was 26 [22–29] Kg/m^2^. The BMI was 27 [23–30] kg/m^2^ among males and 24 [21–28] kg/m^2^ among females. The interobserver variability of the PDFF values was considered to be excellent (ICC = 0.97 [0.94–0.98). Median PDFF values in the subcutaneous fat were 94 [92–95] % yielding an IQR/M of 3%.

### Proton density fat fraction analysis

There was a statistically significant difference in PDFF values between the three ROI locations evaluated (epiphysis, intertrochanteric region, and greater trochanter) (*P* = 0.0001). The highest PDFF values were found in the greater trochanter (median 93 [88–95] %), followed by the epiphysis (median 89 [84–92] %) and finally the intertrochanteric region (median 80 [70–89] %). At the latter location, median PDFF values were 10–14% lower than in the epiphysis and greater trochanter respectively with a higher IQR/M (23.7% at the intertrochanteric region versus 9% and 7.5% at the epiphysis and greater trochanter respectively). PDFF values in the three ROI locations were strongly correlated (p < 0.0001 for each two-by-two correlations)

PDFF value are significantly different between sex in the intertrochanteric region (*P* = 0.0001) and greater trochanter (*P* = 0.005) but not epiphysis (p = 0.071). In all locations, PDFF values were higher in men compared to women (3.3%, 4.4% and 13.1% higher in the epiphysis, greater trochanter and intertrochanteric region respectively). The highest PDFF variation was seen in the intertrochanteric region, with higher variations observed in females (IQR/M = 24.9%) than in males (IQR/M = 14.1-%) with median PDFF values varying from 85 [79–91]% in men and from 75 [65–83]% in women.

**Figure 2:**
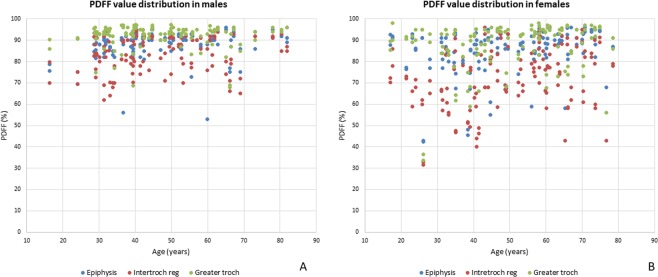
Proton density fat fraction (PDFF) versus age graphs in males (**A**) and females (**B**) in the three locations evaluated. Note the broader distribution of PDFF values in females in all locations and the wider PDFF value distribution in the intertrochanteric region (red dots) compared to the other locations in both males and females.

Patient age was significantly correlated with PDFF value distribution in all regions evaluated (intertrochanteric region *P* = 0.0001, epiphysis *P* = 0.0001 and greater trochanter *P* = 0.024) (Fig. 2). The BMI was not correlated with PDFF values significantly regardless of the location (*P* > 0.50) and no statistical difference was obtained for the side (left versus right) in the 126 patients for whom both hips were available for analysis (*P* > 0.17).

**Figure 3:**
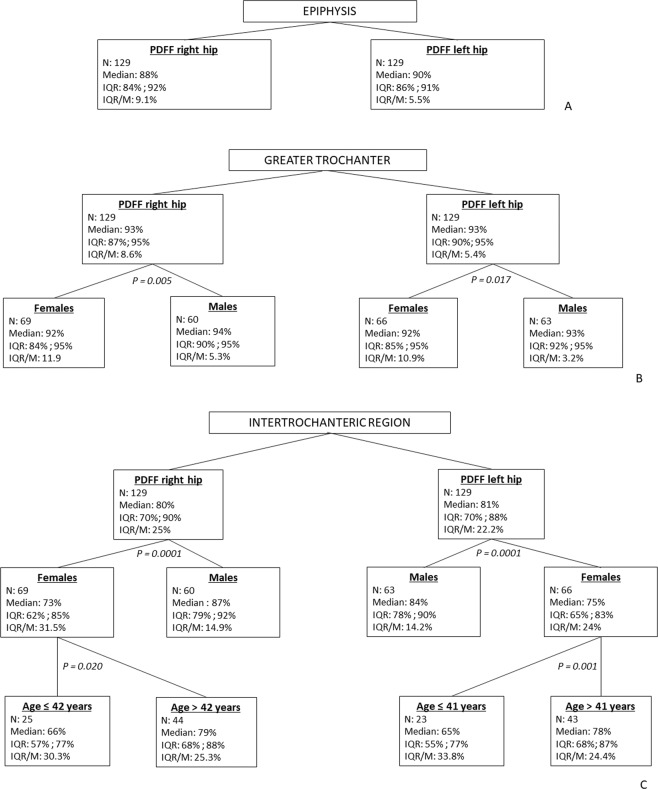
Chi-squared automatic interaction detector (CHAID) decision trees of proton density fat-fraction (PDFF) values of the proximal portion of the femur in the right and left side separately considering sex, age and BMI as variables in the epiphysis (**A**), greater trochanter (**B**) and intertrochanteric region. (**C**) The number of hips in each subgroup (N), PDFF median (M), interquartile range (IQR) and IQR/M are presented. The *P* values of the separation nodes that showed the same tendency in both sides are presented. The only statistically significant influence of BMI (p = 0,014) with a threshold of 29 Kg/m2 was seen in the intertrochanteric region of the right hips of females over 42 years (not shown).

Based on CHAID decision trees, PDFF values in the intertrochanteric region were the most discriminated by the variables with sex (p = 0.0001) appearing more discriminant than age. Age influenced PDFF values only in the female subgroup with a threshold of 41–42 years (*P* = 0.001 and 0.020 in the right and left sides respectively). In the greater trochanter, sex was the only variable to significantly influenced PDFF values on both sides (*P* = 0.005 and 0.017 on the right and left sides respectively). In the epiphysis, none of the variables studied influenced the distribution of PDFF values on both sides (Fig. 3).

Mean PDFF values were invariably lower in the younger subgroups, with the highest variation seen among females in the intertrochanteric region (IQR/M varying from 24–31.5% % in women and 14.2–14.9% in men). Additionally, PDFF variation was higher in females under 41–42 years-old (IQR/M = 30.3–33.8%) compared to the older subgroup (IQR/M = 24.4–25.3%).

BMI had a week influence on PDFF values. This variable only significantly influenced PDFF values in the intertrochanteric region of the right hips of females over 42 years-old. In this subgroup, patients with a BMI higher than 29 presented significantly lower PDFF values than those with lower BMI (median of 61 [54–68]% versus 80 [69–89] %)(P = 0.014).

## Discussion

Patient sex was one of the main factors affecting the distribution of PDFF values in the studied population particularly in the greater trochanter and intertrochanteric region (*P* < 0.005). Bone marrow PDFF was relatively constant in males with a PDFF IQR/M lower than 14%, whereas in females, CV values were as high as 24.9%. Following prior literature reports on vertebral body marrow fat content, an age-related variation in fat content was also found in female femurs^18^. In females under 41–42 years-old, who could be considered to be hormonally active (95% CI for menopause age in developed nations = 48.2–49.2 years), bone marrow fat content was up to 13% lower than that of older females^24^. The variation in PDFF values was also slightly higher in females under 42 years-old (IQR/M = 30.3–33.8% and 24.4–25.3%in younger and older females respectively). Moreover, the distribution of PDFF values found in the study population was similar to that of previous literature reports with an excellent measurement interobserver variability (ICC = 0.97)^25,26^. To the best of our knowledge, this is the largest *in vivo* study evaluating the normal variation in PDFF values at the proximal femur with previous reports being scarce and centred on a limited number of patients in specific subgroups^25–27^. In light of these results, specific standards of reference are needed depending on patient sex and age. The IQR of PDFF values presented in Fig. 3 could be used as such. This data could facilitate future studies assessing proximal femur fat content in pathologic patients.

Measurement location in the proximal femur significantly influenced PDFF values (*P* < 0.0001). Compared to the femoral epiphysis and greater trochanter, PDFF values in the intertrochanteric region was 8–13% lower and more variable (15% versus 16% respectively)^25,28^. Bone marrow fat content is related to the presence of hematopoietic cells, which in turn are linked to bone metabolic activity^29^. The higher variation in fat content found in the inter-trochanteric region could be related to the relatively high hematopoietic marrow content in the proximal femoral metaphysis compared to epiphyseal and apophyseal areas which tend to be predominantly fatty^30^. This difference could be related to other factors not associated with patient sex, such as mechanical stress loading which may influence bone marrow perfusion (and secondarily adiposity) in the femoral epiphysis^31^. Hence, to allow inter-patient comparisons, PDFF assessment should be performed in similar anatomic locations. As hematopoietic/fatty marrow ratio changes related to systemic diseases are first seen in the metaphyseal region, intertrochanteric PDFF measurements are likely to be more sensitive for early bone marrow fat content changes. On the other hand, since bone marrow at the epiphyseal and greater trochanteric regions tend to be affected later in the course of systemic diseases PDFF measurements could be more specific for neoplastic infiltration or carry prognostic implications in cases of marrow deposit diseases^32^.

The BMI had little influence on PDFF values. Females with high BMI tended to have lower PDFF values in the proximal femur, but these findings were only significant in the intertrochanteric region of the right hips of females over 42 years-old (P = 0.014). This finding could be explained by the fact that there is an established relationship between bone metabolism and obesity^33^. Prior studies have also indicated that high BMI values were associated with an increase in bone marrow vascularization, which could also be associated with a reduction in marrow adiposity and consequently PDFF values^34^.

A 12 point IDEAL sequence was used to allow PDFF estimation in this study. This technique is considered to be robust and is widely used for both clinical and research applications. This sequence uses B_0_ and T2* corrections that have been reported improve the accuracy of fat content estimation^14^. Additionally, a low flip angle was used (5°) to limit the influence of T1 effects. The measurements performed in the subcutaneous fat of the included hips showed PDFF values similar to prior literature reports with a small measurement variation (IQR/M = 3%) which is indicative of a good measurement quality^34,35^.

Various limitations of this work need to be acknowledged. The blood workup and densitometry studies of the patients included were not available for analysis due to the retrospective nature of the study. Patient weight and height were not measured by self-reported, which may be a source of bias in estimating the dependency of PDFF on BMI. Bone marrow fat content estimations were not histologically confirmed. Various phantom studies, however, have confirmed the accuracy of PDFF estimation IDEAL sequences^14^. Although a T2* correction was applied, the used sequence was tailored for the control of iron content related bias on hepatic fat content assessment^16,36^. To the best of our knowledge, bone-specific water-fat chemical shift sequences are not yet commercialized. The influence of bone mineral density and clinical and hormonal factors on PDFF values was not evaluated in this study. Further investigation is needed to ascertain the impact of these factors on PDFF measurements. Although BMI significantly influenced PDFF values in the right hip of females over 42 years old, the limited number of hips in this subgroup (six) limited the implications of this finding. Further studies are needed to ascertain the relationship between BMI and PDFF values. The potential technical variation in PDFF values in different MRI scanner models and water-fat chemical shift sequence types was also not assessed in this study.

In conclusion, patient sex, age, and measurement location significantly influenced PDFF values in normal appearing proximal femurs indicating that specific PDFF standards of reference are needed depending on these factors. Median, and IQR of PDFF values in all subgroups evaluated were provided. This data could be used as a reference for future studies on bone marrow fat-content using WFI in the proximal femur until larger, multicentric studies became available.
